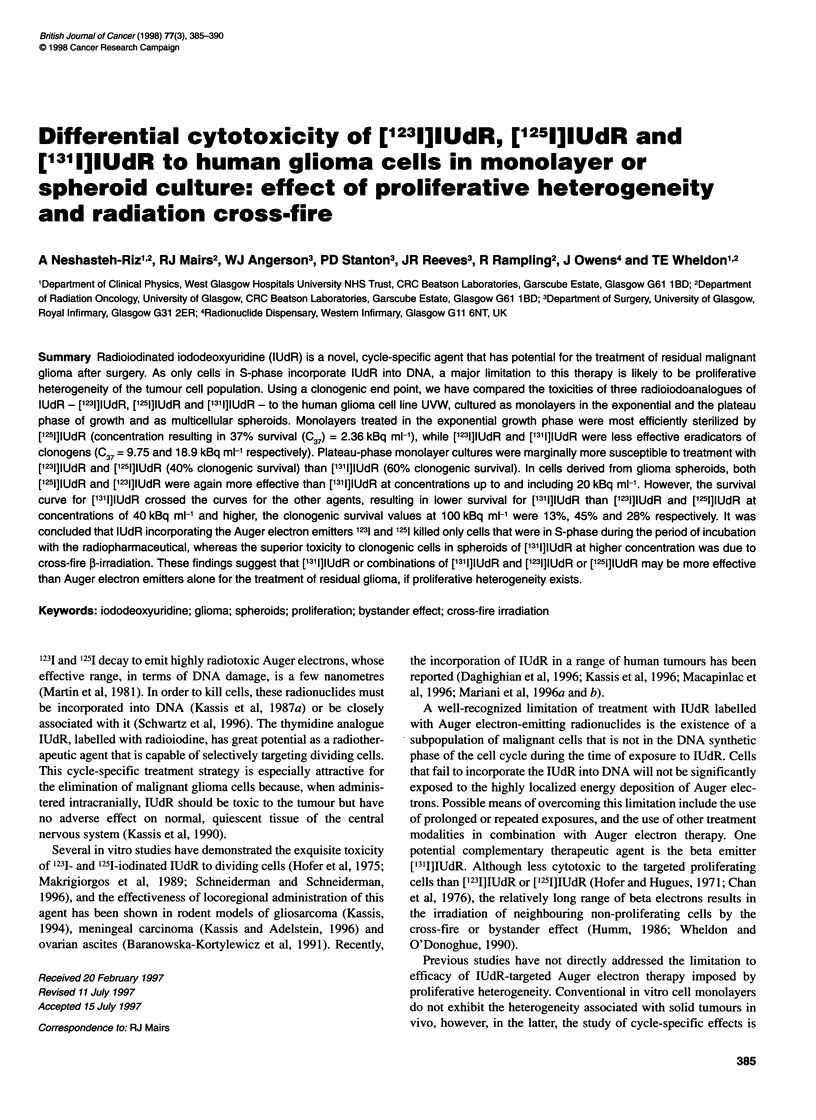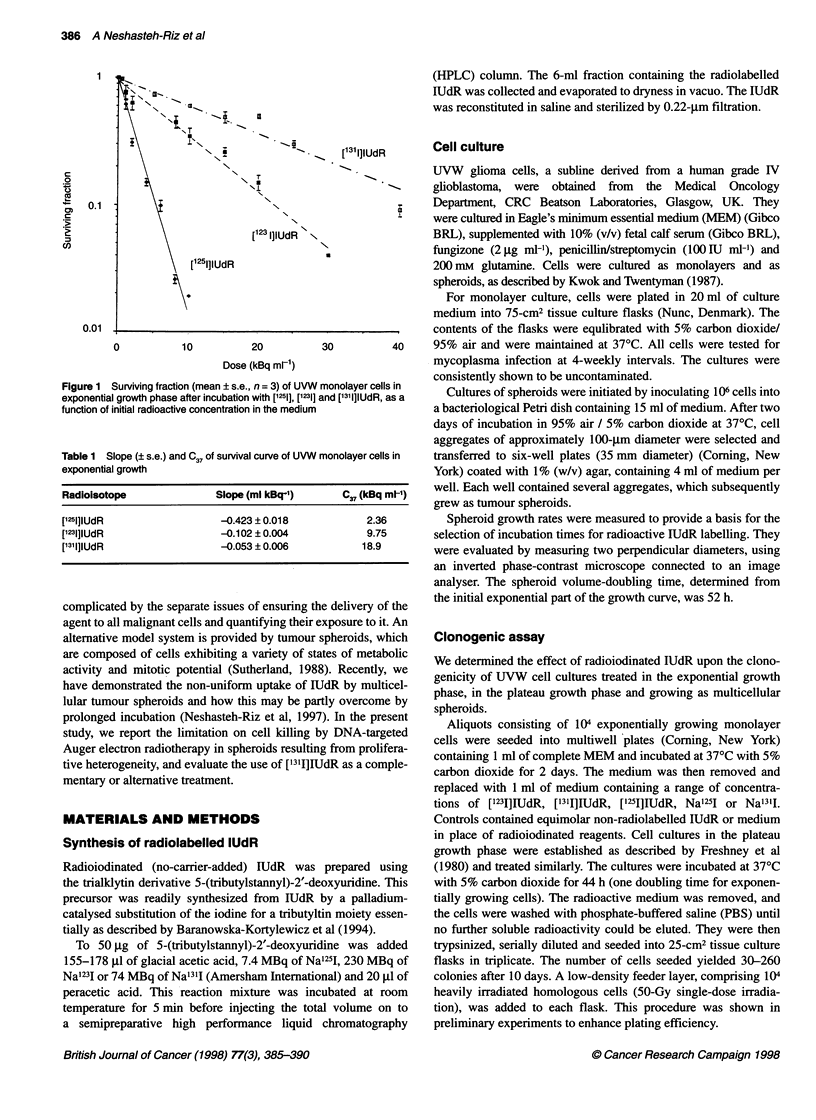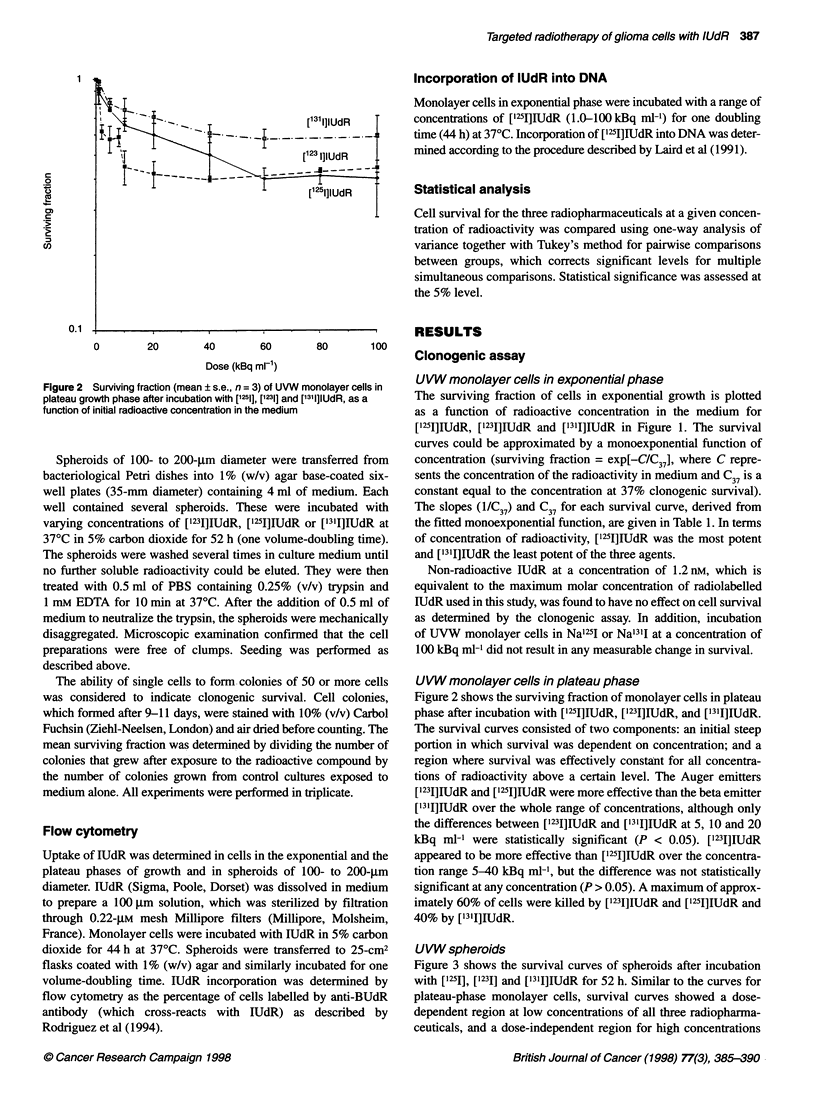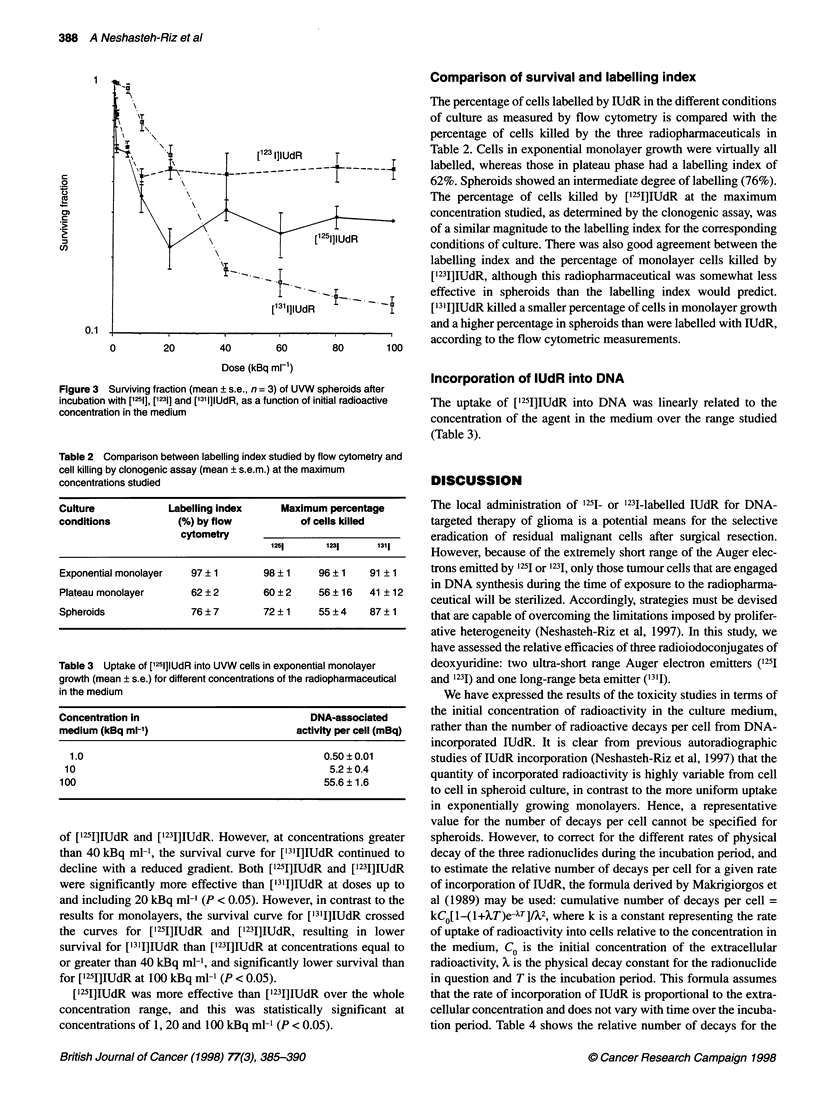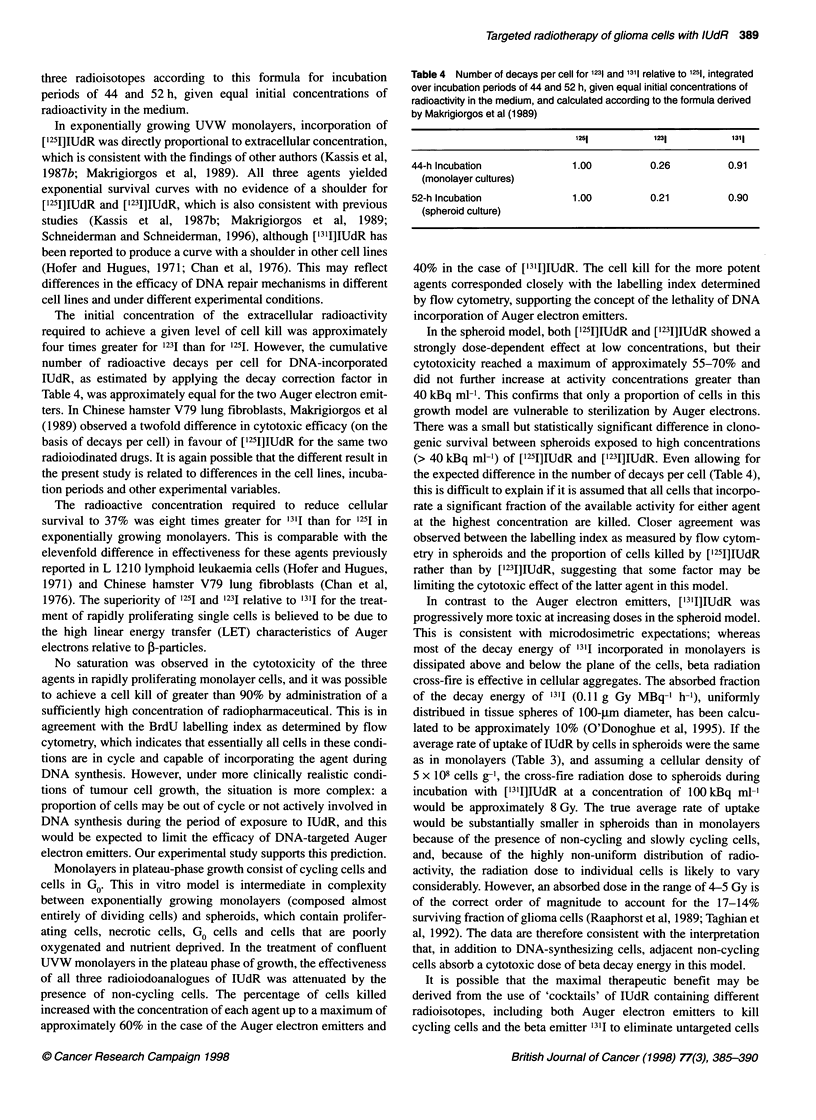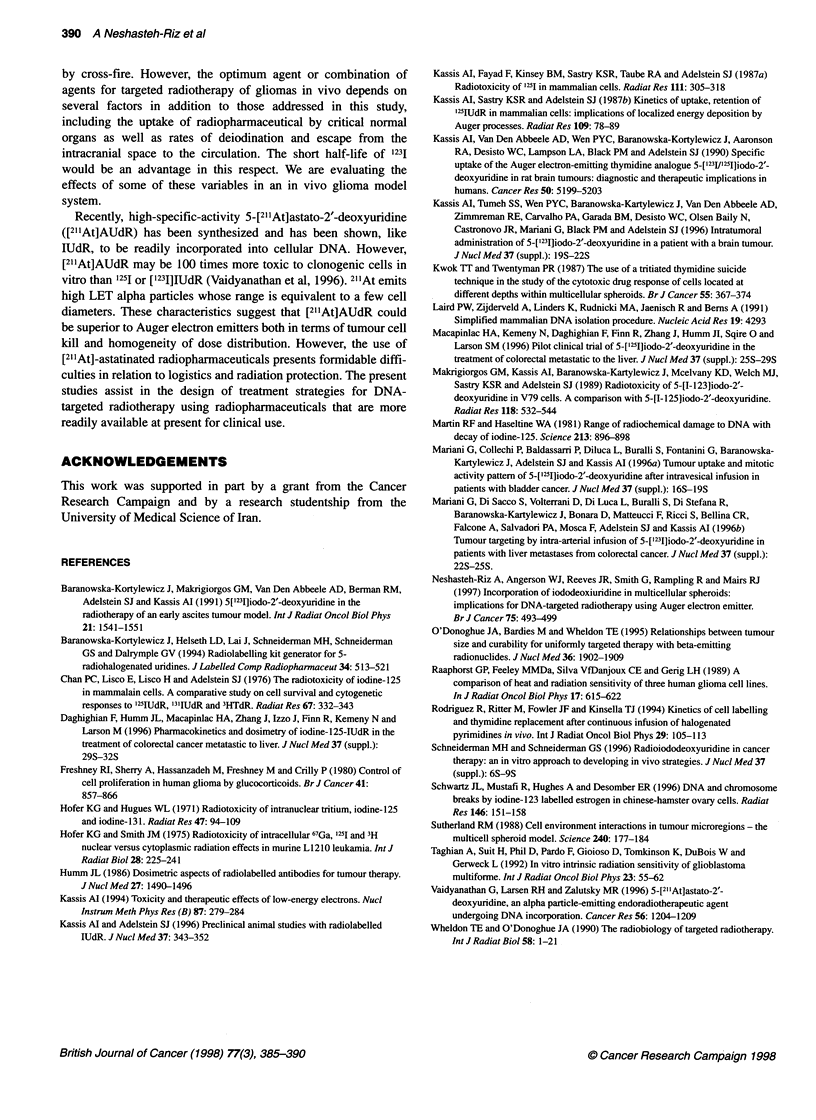# Differential cytotoxicity of [123I]IUdR, [125I]IUdR and [131I]IUdR to human glioma cells in monolayer or spheroid culture: effect of proliferative heterogeneity and radiation cross-fire.

**DOI:** 10.1038/bjc.1998.61

**Published:** 1998

**Authors:** A. Neshasteh-Riz, R. J. Mairs, W. J. Angerson, P. D. Stanton, J. R. Reeves, R. Rampling, J. Owens, T. E. Wheldon

**Affiliations:** Department of Clinical Physics, West Glasgow Hospitals University NHS Trust, CRC Beatson Laboratories, UK.

## Abstract

Radioiodinated iododeoxyuridine (IUdR) is a novel, cycle-specific agent that has potential for the treatment of residual malignant glioma after surgery. As only cells in S-phase incorporate IUdR into DNA, a major limitation to this therapy is likely to be proliferative heterogeneity of the tumour cell population. Using a clonogenic end point, we have compared the toxicities of three radioiodoanalogues of IUdR--[123I]IUdR, [125I]IUdR and [131I]IUdR--to the human glioma cell line UVW, cultured as monolayers in the exponential and the plateau phase of growth and as multicellular spheroids. Monolayers treated in the exponential growth phase were most efficiently sterilized by [125I]IUdR (concentration resulting in 37% survival (C37) = 2.36 kBq ml(-1)), while [123I]IUdR and [131I]IUdR were less effective eradicators of clonogens (C37 = 9.75 and 18.9 kBq ml(-1) respectively). Plateau-phase monolayer cultures were marginally more susceptible to treatment with [123I]IUdR and [125I]IUdR (40% clonogenic survival) than [131I]IUdR (60% clonogenic survival). In cells derived from glioma spheroids, both [125I]IUdR and [123I]IUdR were again more effective than [131I]IUdR at concentrations up to and including 20 kBq ml(-1). However, the survival curve for [131I]IUdR crossed the curves for the other agents, resulting in lower survival for [131I]IUdR than [123I]IUdR and [125I]IUdR at concentrations of 40 kBq ml(-1) and higher, the clonogenic survival values at 100 kBq ml(-1) were 13%, 45% and 28% respectively. It was concluded that IUdR incorporating the Auger electron emitters 123I and 125I killed only cells that were in S-phase during the period of incubation with the radiopharmaceutical, whereas the superior toxicity to clonogenic cells in spheroids of [131I]IUdR at higher concentration was due to cross-fire beta-irradiation. These findings suggest that [131I]IUdR or combinations of [131I]IUdR and [123I]IUdR or [125I]IUdR may be more effective than Auger electron emitters alone for the treatment of residual glioma, if proliferative heterogeneity exists.


British Journal of Cancer (1998) 77(3), 385–390
© 1998 Cancer Research Campaign

### Differential cytotoxicity of [$^{123}$I]IUdR, [$^{125}$I]IUdR and [$^{131}$I]IUdR to human glioma cells in monolayer or spheroid culture: effect of proliferative heterogeneity and radiation cross-fire

**A Neshasteh-Riz[1,2], RJ Mairs[2], WJ Angerson[3], PD Stanton[3], JR Reeves[3], R Rampling[2], J Owens[4] and TE Wheldon[1,2]**

[1]Department of Clinical Physics, West Glasgow Hospitals University NHS Trust, CRC Beatson Laboratories, Garscube Estate, Glasgow G61 1BD; [2]Department of Radiation Oncology, University of Glasgow, CRC Beatson Laboratories, Garscube Estate, Glasgow G61 1BD; [3]Department of Surgery, University of Glasgow, Royal Infirmary, Glasgow G31 2ER; [4]Radionuclide Dispensary, Western Infirmary, Glasgow G11 6NT, UK

**Summary** Radioiodinated iododeoxyuridine (IUdR) is a novel, cycle-specific agent that has potential for the treatment of residual malignant glioma after surgery. As only cells in S-phase incorporate IUdR into DNA, a major limitation to this therapy is likely to be proliferative heterogeneity of the tumour cell population. Using a clonogenic end point, we have compared the toxicities of three radioiodoanalogues of IUdR – [$^{123}$I]IUdR, [$^{125}$I]IUdR and [$^{131}$I]IUdR – to the human glioma cell line UVW, cultured as monolayers in the exponential and the plateau phase of growth and as multicellular spheroids. Monolayers treated in the exponential growth phase were most efficiently sterilized by [$^{125}$I]IUdR (concentration resulting in 37% survival ($C_{37}$) = 2.36 kBq ml$^{-1}$), while [$^{123}$I]IUdR and [$^{131}$I]IUdR were less effective eradicators of clonogens ($C_{37}$ = 9.75 and 18.9 kBq ml$^{-1}$ respectively). Plateau-phase monolayer cultures were marginally more susceptible to treatment with [$^{123}$I]IUdR and [$^{125}$I]IUdR (40% clonogenic survival) than [$^{131}$I]IUdR (60% clonogenic survival). In cells derived from glioma spheroids, both [$^{125}$I]IUdR and [$^{123}$I]IUdR were again more effective than [$^{131}$I]IUdR at concentrations up to and including 20 kBq ml$^{-1}$. However, the survival curve for [$^{131}$I]IUdR crossed the curves for the other agents, resulting in lower survival for [$^{131}$I]IUdR than [$^{123}$I]IUdR and [$^{125}$I]IUdR at concentrations of 40 kBq ml$^{-1}$ and higher, the clonogenic survival values at 100 kBq ml$^{-1}$ were 13%, 45% and 28% respectively. It was concluded that IUdR incorporating the Auger electron emitters $^{123}$I and $^{125}$I killed only cells that were in S-phase during the period of incubation with the radiopharmaceutical, whereas the superior toxicity to clonogenic cells in spheroids of [$^{131}$I]IUdR at higher concentration was due to cross-fire β-irradiation. These findings suggest that [$^{131}$I]IUdR or combinations of [$^{131}$I]IUdR and [$^{123}$I]IUdR or [$^{125}$I]IUdR may be more effective than Auger electron emitters alone for the treatment of residual glioma, if proliferative heterogeneity exists.

**Keywords:** iododeoxyuridine; glioma; spheroids; proliferation; bystander effect; cross-fire irradiation

$^{123}$I and $^{125}$I decay to emit highly radiotoxic Auger electrons, whose effective range, in terms of DNA damage, is a few nanometres (Martin et al, 1981). In order to kill cells, these radionuclides must be incorporated into DNA (Kassis et al, 1987*a*) or be closely associated with it (Schwartz et al, 1996). The thymidine analogue IUdR, labelled with radioiodine, has great potential as a radiotherapeutic agent that is capable of selectively targeting dividing cells. This cycle-specific treatment strategy is especially attractive for the elimination of malignant glioma cells because, when administered intracranially, IUdR should be toxic to the tumour but have no adverse effect on normal, quiescent tissue of the central nervous system (Kassis et al, 1990).

Several in vitro studies have demonstrated the exquisite toxicity of $^{123}$I- and $^{125}$I-iodinated IUdR to dividing cells (Hofer et al, 1975; Makrigiorgos et al, 1989; Schneiderman and Schneiderman, 1996), and the effectiveness of locoregional administration of this agent has been shown in rodent models of gliosarcoma (Kassis, 1994), meningeal carcinoma (Kassis and Adelstein, 1996) and ovarian ascites (Baranowska-Kortylewicz et al, 1991). Recently, the incorporation of IUdR in a range of human tumours has been reported (Daghighian et al, 1996; Kassis et al, 1996; Macapinlac et al, 1996; Mariani et al, 1996*a* and *b*).

A well-recognized limitation of treatment with IUdR labelled with Auger electron-emitting radionuclides is the existence of a subpopulation of malignant cells that is not in the DNA synthetic phase of the cell cycle during the time of exposure to IUdR. Cells that fail to incorporate the IUdR into DNA will not be significantly exposed to the highly localized energy deposition of Auger electrons. Possible means of overcoming this limitation include the use of prolonged or repeated exposures, and the use of other treatment modalities in combination with Auger electron therapy. One potential complementary therapeutic agent is the beta emitter [$^{131}$I]IUdR. Although less cytotoxic to the targeted proliferating cells than [$^{123}$I]IUdR or [$^{125}$I]IUdR (Hofer and Hugues, 1971; Chan et al, 1976), the relatively long range of beta electrons results in the irradiation of neighbouring non-proliferating cells by the cross-fire or bystander effect (Humm, 1986; Wheldon and O'Donoghue, 1990).

Previous studies have not directly addressed the limitation to efficacy of IUdR-targeted Auger electron therapy imposed by proliferative heterogeneity. Conventional in vitro cell monolayers do not exhibit the heterogeneity associated with solid tumours in vivo, however, in the latter, the study of cycle-specific effects is

*Received 20 February 1997*
*Revised 11 July 1997*
*Accepted 15 July 1997*

*Correspondence to:* RJ Mairs

**Figure 1** Surviving fraction (mean ± s.e., $n = 3$) of UVW monolayer cells in exponential growth phase after incubation with [$^{125}$I], [$^{123}$I] and [$^{131}$I]IUdR, as a function of initial radioactive concentration in the medium

**Table 1** Slope (± s.e.) and $C_{37}$ of survival curve of UVW monolayer cells in exponential growth

| Radioisotope | Slope (ml kBq$^{-1}$) | $C_{37}$ (kBq ml$^{-1}$) |
|---|---|---|
| [$^{125}$I]IUdR | –0.423 ± 0.018 | 2.36 |
| [$^{123}$I]IUdR | –0.102 ± 0.004 | 9.75 |
| [$^{131}$I]IUdR | –0.053 ± 0.006 | 18.9 |

complicated by the separate issues of ensuring the delivery of the agent to all malignant cells and quantifying their exposure to it. An alternative model system is provided by tumour spheroids, which are composed of cells exhibiting a variety of states of metabolic activity and mitotic potential (Sutherland, 1988). Recently, we have demonstrated the non-uniform uptake of IUdR by multicellular tumour spheroids and how this may be partly overcome by prolonged incubation (Neshasteh-Riz et al, 1997). In the present study, we report the limitation on cell killing by DNA-targeted Auger electron radiotherapy in spheroids resulting from proliferative heterogeneity, and evaluate the use of [$^{131}$I]IUdR as a complementary or alternative treatment.

### MATERIALS AND METHODS

### Synthesis of radiolabelled IUdR

Radioiodinated (no-carrier-added) IUdR was prepared using the trialklytin derivative 5-(tributylstannyl)-2′-deoxyuridine. This precursor was readily synthesized from IUdR by a palladium-catalysed substitution of the iodine for a tributyltin moiety essentially as described by Baranowska-Kortylewicz et al (1994).

To 50 µg of 5-(tributylstannyl)-2′-deoxyuridine was added 155–178 µl of glacial acetic acid, 7.4 MBq of Na$^{125}$I, 230 MBq of Na$^{123}$I or 74 MBq of Na$^{131}$I (Amersham International) and 20 µl of peracetic acid. This reaction mixture was incubated at room temperature for 5 min before injecting the total volume on to a semipreparative high performance liquid chromatography (HPLC) column. The 6-ml fraction containing the radiolabelled IUdR was collected and evaporated to dryness in vacuo. The IUdR was reconstituted in saline and sterilized by 0.22-µm filtration.

### Cell culture

UVW glioma cells, a subline derived from a human grade IV glioblastoma, were obtained from the Medical Oncology Department, CRC Beatson Laboratories, Glasgow, UK. They were cultured in Eagle's minimum essential medium (MEM) (Gibco BRL), supplemented with 10% (v/v) fetal calf serum (Gibco BRL), fungizone (2 µg ml$^{-1}$), penicillin/streptomycin (100 IU ml$^{-1}$) and 200 mM glutamine. Cells were cultured as monolayers and as spheroids, as described by Kwok and Twentyman (1987).

For monolayer culture, cells were plated in 20 ml of culture medium into 75-cm$^2$ tissue culture flasks (Nunc, Denmark). The contents of the flasks were equlibrated with 5% carbon dioxide/ 95% air and were maintained at 37°C. All cells were tested for mycoplasma infection at 4-weekly intervals. The cultures were consistently shown to be uncontaminated.

Cultures of spheroids were initiated by inoculating $10^6$ cells into a bacteriological Petri dish containing 15 ml of medium. After two days of incubation in 95% air / 5% carbon dioxide at 37°C, cell aggregates of approximately 100-µm diameter were selected and transferred to six-well plates (35 mm diameter) (Corning, New York) coated with 1% (w/v) agar, containing 4 ml of medium per well. Each well contained several aggregates, which subsequently grew as tumour spheroids.

Spheroid growth rates were measured to provide a basis for the selection of incubation times for radioactive IUdR labelling. They were evaluated by measuring two perpendicular diameters, using an inverted phase-contrast microscope connected to an image analyser. The spheroid volume-doubling time, determined from the initial exponential part of the growth curve, was 52 h.

### Clonogenic assay

We determined the effect of radioiodinated IUdR upon the clonogenicity of UVW cell cultures treated in the exponential growth phase, in the plateau growth phase and growing as multicellular spheroids.

Aliquots consisting of $10^4$ exponentially growing monolayer cells were seeded into multiwell plates (Corning, New York) containing 1 ml of complete MEM and incubated at 37°C with 5% carbon dioxide for 2 days. The medium was then removed and replaced with 1 ml of medium containing a range of concentrations of [$^{123}$I]IUdR, [$^{131}$I]IUdR, [$^{125}$I]IUdR, Na$^{125}$I or Na$^{131}$I. Controls contained equimolar non-radiolabelled IUdR or medium in place of radioiodinated reagents. Cell cultures in the plateau growth phase were established as described by Freshney et al (1980) and treated similarly. The cultures were incubated at 37°C with 5% carbon dioxide for 44 h (one doubling time for exponentially growing cells). The radioactive medium was removed, and the cells were washed with phosphate-buffered saline (PBS) until no further soluble radioactivity could be eluted. They were then trypsinized, serially diluted and seeded into 25-cm$^2$ tissue culture flasks in triplicate. The number of cells seeded yielded 30–260 colonies after 10 days. A low-density feeder layer, comprising $10^4$ heavily irradiated homologous cells (50-Gy single-dose irradiation), was added to each flask. This procedure was shown in preliminary experiments to enhance plating efficiency.

© Cancer Research Campaign 1998

**Figure 2** Surviving fraction (mean ± s.e., $n = 3$) of UVW monolayer cells in plateau growth phase after incubation with [$^{125}$I], [$^{123}$I] and [$^{131}$I]IUdR, as a function of initial radioactive concentration in the medium

Spheroids of 100- to 200-μm diameter were transferred from bacteriological Petri dishes into 1% (w/v) agar base-coated six-well plates (35-mm diameter) containing 4 ml of medium. Each well contained several spheroids. These were incubated with varying concentrations of [$^{123}$I]IUdR, [$^{125}$I]IUdR or [$^{131}$I]IUdR at 37°C in 5% carbon dioxide for 52 h (one volume-doubling time). The spheroids were washed several times in culture medium until no further soluble radioactivity could be eluted. They were then treated with 0.5 ml of PBS containing 0.25% (v/v) trypsin and 1 mM EDTA for 10 min at 37°C. After the addition of 0.5 ml of medium to neutralize the trypsin, the spheroids were mechanically disaggregated. Microscopic examination confirmed that the cell preparations were free of clumps. Seeding was performed as described above.

The ability of single cells to form colonies of 50 or more cells was considered to indicate clonogenic survival. Cell colonies, which formed after 9–11 days, were stained with 10% (v/v) Carbol Fuchsin (Ziehl-Neelsen, London) and air dried before counting. The mean surviving fraction was determined by dividing the number of colonies that grew after exposure to the radioactive compound by the number of colonies grown from control cultures exposed to medium alone. All experiments were performed in triplicate.

### Flow cytometry

Uptake of IUdR was determined in cells in the exponential and the plateau phases of growth and in spheroids of 100- to 200-μm diameter. IUdR (Sigma, Poole, Dorset) was dissolved in medium to prepare a 100 μm solution, which was sterilized by filtration through 0.22-μM mesh Millipore filters (Millipore, Molsheim, France). Monolayer cells were incubated with IUdR in 5% carbon dioxide for 44 h at 37°C. Spheroids were transferred to 25-cm$^2$ flasks coated with 1% (w/v) agar and similarly incubated for one volume-doubling time. IUdR incorporation was determined by flow cytometry as the percentage of cells labelled by anti-BUdR antibody (which cross-reacts with IUdR) as described by Rodriguez et al (1994).

### Incorporation of IUdR into DNA

Monolayer cells in exponential phase were incubated with a range of concentrations of [$^{125}$I]IUdR (1.0–100 kBq ml$^{-1}$) for one doubling time (44 h) at 37°C. Incorporation of [$^{125}$I]IUdR into DNA was determined according to the procedure described by Laird et al (1991).

### Statistical analysis

Cell survival for the three radiopharmaceuticals at a given concentration of radioactivity was compared using one-way analysis of variance together with Tukey's method for pairwise comparisons between groups, which corrects significant levels for multiple simultaneous comparisons. Statistical significance was assessed at the 5% level.

### RESULTS

### Clonogenic assay

### *UVW monolayer cells in exponential phase*

The surviving fraction of cells in exponential growth is plotted as a function of radioactive concentration in the medium for [$^{125}$I]IUdR, [$^{123}$I]IUdR and [$^{131}$I]IUdR in Figure 1. The survival curves could be approximated by a monoexponential function of concentration (surviving fraction = $\exp[-C/C_{37}]$, where $C$ represents the concentration of the radioactivity in medium and $C_{37}$ is a constant equal to the concentration at 37% clonogenic survival). The slopes ($1/C_{37}$) and $C_{37}$ for each survival curve, derived from the fitted monoexponential function, are given in Table 1. In terms of concentration of radioactivity, [$^{125}$I]IUdR was the most potent and [$^{131}$I]IUdR the least potent of the three agents.

Non-radioactive IUdR at a concentration of 1.2 nM, which is equivalent to the maximum molar concentration of radiolabelled IUdR used in this study, was found to have no effect on cell survival as determined by the clonogenic assay. In addition, incubation of UVW monolayer cells in Na$^{125}$I or Na$^{131}$I at a concentration of 100 kBq ml$^{-1}$ did not result in any measurable change in survival.

### *UVW monolayer cells in plateau phase*

Figure 2 shows the surviving fraction of monolayer cells in plateau phase after incubation with [$^{125}$I]IUdR, [$^{123}$I]IUdR, and [$^{131}$I]IUdR. The survival curves consisted of two components: an initial steep portion in which survival was dependent on concentration; and a region where survival was effectively constant for all concentrations of radioactivity above a certain level. The Auger emitters [$^{123}$I]IUdR and [$^{125}$I]IUdR were more effective than the beta emitter [$^{131}$I]IUdR over the whole range of concentrations, although only the differences between [$^{123}$I]IUdR and [$^{131}$I]IUdR at 5, 10 and 20 kBq ml$^{-1}$ were statistically significant ($P < 0.05$). [$^{123}$I]IUdR appeared to be more effective than [$^{125}$I]IUdR over the concentration range 5–40 kBq ml$^{-1}$, but the difference was not statistically significant at any concentration ($P > 0.05$). A maximum of approximately 60% of cells were killed by [$^{123}$I]IUdR and [$^{125}$I]IUdR and 40% by [$^{131}$I]IUdR.

### *UVW spheroids*

Figure 3 shows the survival curves of spheroids after incubation with [$^{125}$I], [$^{123}$I] and [$^{131}$I]IUdR for 52 h. Similar to the curves for plateau-phase monolayer cells, survival curves showed a dose-dependent region at low concentrations of all three radiopharmaceuticals, and a dose-independent region for high concentrations

© Cancer Research Campaign 1998

**Figure 3** Surviving fraction (mean ± s.e., $n$ = 3) of UVW spheroids after incubation with [$^{125}$I], [$^{123}$I] and [$^{131}$I]IUdR, as a function of initial radioactive concentration in the medium

**Table 2** Comparison between labelling index studied by flow cytometry and cell killing by clonogenic assay (mean ± s.e.m.) at the maximum concentrations studied

| Culture conditions | Labelling index (%) by flow cytometry | Maximum percentage of cells killed | | |
|---|---|---|---|---|
| | | $^{125}$I | $^{123}$I | $^{131}$I |
| Exponential monolayer | 97 ± 1 | 98 ± 1 | 96 ± 1 | 91 ± 1 |
| Plateau monolayer | 62 ± 2 | 60 ± 2 | 56 ± 16 | 41 ± 12 |
| Spheroids | 76 ± 7 | 72 ± 1 | 55 ± 4 | 87 ± 1 |

**Table 3** Uptake of [$^{125}$I]IUdR into UVW cells in exponential monolayer growth (mean ± s.e.) for different concentrations of the radiopharmaceutical in the medium

| Concentration in medium (kBq ml$^{-1}$) | DNA-associated activity per cell (mBq) |
|---|---|
| 1.0 | 0.50 ± 0.01 |
| 10 | 5.2 ± 0.4 |
| 100 | 55.6 ± 1.6 |

of [$^{125}$I]IUdR and [$^{123}$I]IUdR. However, at concentrations greater than 40 kBq ml$^{-1}$, the survival curve for [$^{131}$I]IUdR continued to decline with a reduced gradient. Both [$^{125}$I]IUdR and [$^{123}$I]IUdR were significantly more effective than [$^{131}$I]IUdR at doses up to and including 20 kBq ml$^{-1}$ ($P < 0.05$). However, in contrast to the results for monolayers, the survival curve for [$^{131}$I]IUdR crossed the curves for [$^{125}$I]IUdR and [$^{123}$I]IUdR, resulting in lower survival for [$^{131}$I]IUdR than [$^{123}$I]IUdR at concentrations equal to or greater than 40 kBq ml$^{-1}$, and significantly lower survival than for [$^{125}$I]IUdR at 100 kBq ml$^{-1}$ ($P < 0.05$).

[$^{125}$I]IUdR was more effective than [$^{123}$I]IUdR over the whole concentration range, and this was statistically significant at concentrations of 1, 20 and 100 kBq ml$^{-1}$ ($P < 0.05$).

### Comparison of survival and labelling index

The percentage of cells labelled by IUdR in the different conditions of culture as measured by flow cytometry is compared with the percentage of cells killed by the three radiopharmaceuticals in Table 2. Cells in exponential monolayer growth were virtually all labelled, whereas those in plateau phase had a labelling index of 62%. Spheroids showed an intermediate degree of labelling (76%). The percentage of cells killed by [$^{125}$I]IUdR at the maximum concentration studied, as determined by the clonogenic assay, was of a similar magnitude to the labelling index for the corresponding conditions of culture. There was also good agreement between the labelling index and the percentage of monolayer cells killed by [$^{123}$I]IUdR, although this radiopharmaceutical was somewhat less effective in spheroids than the labelling index would predict. [$^{131}$I]IUdR killed a smaller percentage of cells in monolayer growth and a higher percentage in spheroids than were labelled with IUdR, according to the flow cytometric measurements.

### Incorporation of IUdR into DNA

The uptake of [$^{125}$I]IUdR into DNA was linearly related to the concentration of the agent in the medium over the range studied (Table 3).

### DISCUSSION

The local administration of $^{125}$I- or $^{123}$I-labelled IUdR for DNA-targeted therapy of glioma is a potential means for the selective eradication of residual malignant cells after surgical resection. However, because of the extremely short range of the Auger electrons emitted by $^{125}$I or $^{123}$I, only those tumour cells that are engaged in DNA synthesis during the time of exposure to the radiopharmaceutical will be sterilized. Accordingly, strategies must be devised that are capable of overcoming the limitations imposed by proliferative heterogeneity (Neshasteh-Riz et al, 1997). In this study, we have assessed the relative efficacies of three radioiodoconjugates of deoxyuridine: two ultra-short range Auger electron emitters ($^{125}$I and $^{123}$I) and one long-range beta emitter ($^{131}$I).

We have expressed the results of the toxicity studies in terms of the initial concentration of radioactivity in the culture medium, rather than the number of radioactive decays per cell from DNA-incorporated IUdR. It is clear from previous autoradiographic studies of IUdR incorporation (Neshasteh-Riz et al, 1997) that the quantity of incorporated radioactivity is highly variable from cell to cell in spheroid culture, in contrast to the more uniform uptake in exponentially growing monolayers. Hence, a representative value for the number of decays per cell cannot be specified for spheroids. However, to correct for the different rates of physical decay of the three radionuclides during the incubation period, and to estimate the relative number of decays per cell for a given rate of incorporation of IUdR, the formula derived by Makrigiorgos et al (1989) may be used: cumulative number of decays per cell = $kC_0[1-(1+\lambda T)e^{-\lambda T}]/\lambda^2$, where k is a constant representing the rate of uptake of radioactivity into cells relative to the concentration in the medium, $C_0$ is the initial concentration of the extracellular radioactivity, $\lambda$ is the physical decay constant for the radionuclide in question and $T$ is the incubation period. This formula assumes that the rate of incorporation of IUdR is proportional to the extracellular concentration and does not vary with time over the incubation period. Table 4 shows the relative number of decays for the

© Cancer Research Campaign 1998

three radioisotopes according to this formula for incubation periods of 44 and 52 h, given equal initial concentrations of radioactivity in the medium.

In exponentially growing UVW monolayers, incorporation of $[^{125}I]$IUdR was directly proportional to extracellular concentration, which is consistent with the findings of other authors (Kassis et al, 1987*b*; Makrigiorgos et al, 1989). All three agents yielded exponential survival curves with no evidence of a shoulder for $[^{125}I]$IUdR and $[^{123}I]$IUdR, which is also consistent with previous studies (Kassis et al, 1987b; Makrigiorgos et al, 1989; Schneiderman and Schneiderman, 1996), although $[^{131}I]$IUdR has been reported to produce a curve with a shoulder in other cell lines (Hofer and Hugues, 1971; Chan et al, 1976). This may reflect differences in the efficacy of DNA repair mechanisms in different cell lines and under different experimental conditions.

The initial concentration of the extracellular radioactivity required to achieve a given level of cell kill was approximately four times greater for $^{123}I$ than for $^{125}I$. However, the cumulative number of radioactive decays per cell for DNA-incorporated IUdR, as estimated by applying the decay correction factor in Table 4, was approximately equal for the two Auger electron emitters. In Chinese hamster V79 lung fibroblasts, Makrigiorgos et al (1989) observed a twofold difference in cytotoxic efficacy (on the basis of decays per cell) in favour of $[^{125}I]$IUdR for the same two radioiodinated drugs. It is again possible that the different result in the present study is related to differences in the cell lines, incubation periods and other experimental variables.

The radioactive concentration required to reduce cellular survival to 37% was eight times greater for $^{131}I$ than for $^{125}I$ in exponentially growing monolayers. This is comparable with the elevenfold difference in effectiveness for these agents previously reported in L 1210 lymphoid leukaemia cells (Hofer and Hugues, 1971) and Chinese hamster V79 lung fibroblasts (Chan et al, 1976). The superiority of $^{125}I$ and $^{123}I$ relative to $^{131}I$ for the treatment of rapidly proliferating single cells is believed to be due to the high linear energy transfer (LET) characteristics of Auger electrons relative to β-particles.

No saturation was observed in the cytotoxicity of the three agents in rapidly proliferating monolayer cells, and it was possible to achieve a cell kill of greater than 90% by administration of a sufficiently high concentration of radiopharmaceutical. This is in agreement with the BrdU labelling index as determined by flow cytometry, which indicates that essentially all cells in these conditions are in cycle and capable of incorporating the agent during DNA synthesis. However, under more clinically realistic conditions of tumour cell growth, the situation is more complex: a proportion of cells may be out of cycle or not actively involved in DNA synthesis during the period of exposure to IUdR, and this would be expected to limit the efficacy of DNA-targeted Auger electron emitters. Our experimental study supports this prediction.

Monolayers in plateau-phase growth consist of cycling cells and cells in $G_0$. This in vitro model is intermediate in complexity between exponentially growing monolayers (composed almost entirely of dividing cells) and spheroids, which contain proliferating cells, necrotic cells, $G_0$ cells and cells that are poorly oxygenated and nutrient deprived. In the treatment of confluent UVW monolayers in the plateau phase of growth, the effectiveness of all three radioiodoanalogues of IUdR was attenuated by the presence of non-cycling cells. The percentage of cells killed increased with the concentration of each agent up to a maximum of approximately 60% in the case of the Auger electron emitters and 40% in the case of $[^{131}I]$IUdR. The cell kill for the more potent agents corresponded closely with the labelling index determined by flow cytometry, supporting the concept of the lethality of DNA incorporation of Auger electron emitters.

**Table 4** Number of decays per cell for $^{123}I$ and $^{131}I$ relative to $^{125}I$, integrated over incubation periods of 44 and 52 h, given equal initial concentrations of radioactivity in the medium, and calculated according to the formula derived by Makrigiorgos et al (1989)

| | $^{125}I$ | $^{123}I$ | $^{131}I$ |
|---|---|---|---|
| 44-h Incubation (monolayer cultures) | 1.00 | 0.26 | 0.91 |
| 52-h Incubation (spheroid culture) | 1.00 | 0.21 | 0.90 |

In the spheroid model, both $[^{125}I]$IUdR and $[^{123}I]$IUdR showed a strongly dose-dependent effect at low concentrations, but their cytotoxicity reached a maximum of approximately 55–70% and did not further increase at activity concentrations greater than 40 kBq $ml^{-1}$. This confirms that only a proportion of cells in this growth model are vulnerable to sterilization by Auger electrons. There was a small but statistically significant difference in clonogenic survival between spheroids exposed to high concentrations (> 40 kBq $ml^{-1}$) of $[^{125}I]$IUdR and $[^{123}I]$IUdR. Even allowing for the expected difference in the number of decays per cell (Table 4), this is difficult to explain if it is assumed that all cells that incorporate a significant fraction of the available activity for either agent at the highest concentration are killed. Closer agreement was observed between the labelling index as measured by flow cytometry in spheroids and the proportion of cells killed by $[^{125}I]$IUdR rather than by $[^{123}I]$IUdR, suggesting that some factor may be limiting the cytotoxic effect of the latter agent in this model.

In contrast to the Auger electron emitters, $[^{131}I]$IUdR was progressively more toxic at increasing doses in the spheroid model. This is consistent with microdosimetric expectations; whereas most of the decay energy of $^{131}I$ incorporated in monolayers is dissipated above and below the plane of the cells, beta radiation cross-fire is effective in cellular aggregates. The absorbed fraction of the decay energy of $^{131}I$ (0.11 g Gy $MBq^{-1}$ $h^{-1}$), uniformly distribued in tissue spheres of 100-μm diameter, has been calculated to be approximately 10% (O'Donoghue et al, 1995). If the average rate of uptake of IUdR by cells in spheroids were the same as in monolayers (Table 3), and assuming a cellular density of $5 \times 10^8$ cells $g^{-1}$, the cross-fire radiation dose to spheroids during incubation with $[^{131}I]$IUdR at a concentration of 100 kBq $ml^{-1}$ would be approximately 8 Gy. The true average rate of uptake would be substantially smaller in spheroids than in monolayers because of the presence of non-cycling and slowly cycling cells, and, because of the highly non-uniform distribution of radioactivity, the radiation dose to individual cells is likely to vary considerably. However, an absorbed dose in the range of 4–5 Gy is of the correct order of magnitude to account for the 17–14% surviving fraction of glioma cells (Raaphorst et al, 1989; Taghian et al, 1992). The data are therefore consistent with the interpretation that, in addition to DNA-synthesizing cells, adjacent non-cycling cells absorb a cytotoxic dose of beta decay energy in this model.

It is possible that the maximal therapeutic benefit may be derived from the use of 'cocktails' of IUdR containing different radioisotopes, including both Auger electron emitters to kill cycling cells and the beta emitter $^{131}I$ to eliminate untargeted cells

*© Cancer Research Campaign 1998*

by cross-fire. However, the optimum agent or combination of agents for targeted radiotherapy of gliomas in vivo depends on several factors in addition to those addressed in this study, including the uptake of radiopharmaceutical by critical normal organs as well as rates of deiodination and escape from the intracranial space to the circulation. The short half-life of $^{123}$I would be an advantage in this respect. We are evaluating the effects of some of these variables in an in vivo glioma model system.

Recently, high-specific-activity 5-[$^{211}$At]astato-2′-deoxyuridine ([$^{211}$At]AUdR) has been synthesized and has been shown, like IUdR, to be readily incorporated into cellular DNA. However, [$^{211}$At]AUdR may be 100 times more toxic to clonogenic cells in vitro than $^{125}$I or [$^{123}$I]IUdR (Vaidyanathan et al, 1996). $^{211}$At emits high LET alpha particles whose range is equivalent to a few cell diameters. These characteristics suggest that [$^{211}$At]AUdR could be superior to Auger electron emitters both in terms of tumour cell kill and homogeneity of dose distribution. However, the use of [$^{211}$At]-astatinated radiopharmaceuticals presents formidable difficulties in relation to logistics and radiation protection. The present studies assist in the design of treatment strategies for DNA-targeted radiotherapy using radiopharmaceuticals that are more readily available at present for clinical use.

### ACKNOWLEDGEMENTS

This work was supported in part by a grant from the Cancer Research Campaign and by a research studentship from the University of Medical Science of Iran.

### REFERENCES

Baranowska-Kortylewicz J, Makrigiorgos GM, Van Den Abbeele AD, Berman RM, Adelstein SJ and Kassis AI (1991) 5[$^{123}$I]iodo-2′-deoxyuridine in the radiotherapy of an early ascites tumour model. *Int J Radiat Oncol Biol Phys* **21**: 1541–1551

Baranowska-Kortylewicz J, Helseth LD, Lai J, Schneiderman MH, Schneiderman GS and Dalrymple GV (1994) Radiolabelling kit generator for 5-radiohalogenated uridines. *J Labelled Comp Radiopharmaceut* **34**: 513–521

Chan PC, Lisco E, Lisco H and Adelstein SJ (1976) The radiotoxicity of iodine-125 in mammalain cells. A comparative study on cell survival and cytogenetic responses to $^{125}$IUdR, $^{131}$IUdR and $^{3}$HTdR. *Radiat Res* **67**: 332–343

Daghighian F, Humm JL, Macapinlac HA, Zhang J, Izzo J, Finn R, Kemeny N and Larson M (1996) Pharmacokinetics and dosimetry of iodine-125-IUdR in the treatment of colorectal cancer metatastic to liver. *J Nucl Med* **37** (suppl.): 29S–32S

Freshney RI, Sherry A, Hassanzadeh M, Freshney M and Crilly P (1980) Control of cell proliferation in human glioma by glucocorticoids. *Br J Cancer* **41**: 857–866

Hofer KG and Hugues WL (1971) Radiotoxicity of intranuclear tritium, iodine-125 and iodine-131. *Radiat Res* **47**: 94–109

Hofer KG and Smith JM (1975) Radiotoxicity of intracellular $^{67}$Ga, $^{125}$I and $^{3}$H nuclear versus cytoplasmic radiation effects in murine L1210 leukamia. *Int J Radiat Biol* **28**: 225–241

Humm JL (1986) Dosimetric aspects of radiolabelled antibodies for tumour therapy. *J Nucl Med* **27**: 1490–1496

Kassis AI (1994) Toxicity and therapeutic effects of low-energy electrons. *Nucl Instrum Meth Phys Res (B)* **87**: 279–284

Kassis AI and Adelstein SJ (1996) Preclinical animal studies with radiolabelled IUdR. *J Nucl Med* **37**: 343–352

Kassis AI, Fayad F, Kinsey BM, Sastry KSR, Taube RA and Adelstein SJ (1987*a*) Radiotoxicity of $^{125}$I in mammalian cells. *Radiat Res* **111**: 305–318

Kassis AI, Sastry KSR and Adelstein SJ (1987*b*) Kinetics of uptake, retention of $^{125}$IUdR in mammalian cells: implications of localized energy deposition by Auger processes. *Radiat Res* **109**: 78–89

Kassis AI, Van Den Abbeele AD, Wen PYC, Baranowska-Kortylewicz J, Aaronson RA, Desisto WC, Lampson LA, Black PM and Adelstein SJ (1990) Specific uptake of the Auger electron-emitting thymidine analogue 5-[$^{123}$I/$^{125}$I]iodo-2′-deoxyuridine in rat brain tumours: diagnostic and therapeutic implications in humans. *Cancer Res* **50**: 5199–5203

Kassis AI, Tumeh SS, Wen PYC, Baranowska-Kartylewicz J, Van Den Abbeele AD, Zimmreman RE, Carvalho PA, Garada BM, Desisto WC, Olsen Baily N, Castronovo JR, Mariani G, Black PM and Adelstein SJ (1996) Intratumoral administration of 5-[$^{123}$I]iodo-2′-deoxyuridine in a patient with a brain tumour. *J Nucl Med* **37** (suppl.): 19S–22S

Kwok TT and Twentyman PR (1987) The use of a tritiated thymidine suicide technique in the study of the cytotoxic drug response of cells located at different depths within multicellular spheroids. *Br J Cancer* **55**: 367–374

Laird PW, Zijderveld A, Linders K, Rudnicki MA, Jaenisch R and Berns A (1991) Simplified mammalian DNA isolation procedure. *Nucleic Acid Res* **19**: 4293

Macapinlac HA, Kemeny N, Daghighian F, Finn R, Zhang J, Humm JI, Sqire O and Larson SM (1996) Pilot clinical trial of 5-[$^{125}$I]iodo-2′-deoxyuridine in the treatment of colorectal metastatic to the liver. *J Nucl Med* **37** (suppl.): 25S–29S

Makrigiorgos GM, Kassis AI, Baranowska-Kartylewicz J, Mcelvany KD, Welch MJ, Sastry KSR and Adelstein SJ (1989) Radiotoxicity of 5-[I-123]iodo-2′-deoxyuridine in V79 cells. A comparison with 5-[I-125]iodo-2′-deoxyuridine. *Radiat Res* **118**: 532–544

Martin RF and Haseltine WA (1981) Range of radiochemical damage to DNA with decay of iodine-125. *Science* **213**: 896–898

Mariani G, Collechi P, Baldassarri P, Diluca L, Buralli S, Fontanini G, Baranowska-Kartylewicz J, Adelstein SJ and Kassis AI (1996*a*) Tumour uptake and mitotic activity pattern of 5-[$^{125}$I]iodo-2′-deoxyuridine after intravesical infusion in patients with bladder cancer. *J Nucl Med* **37** (suppl.): 16S–19S

Mariani G, Di Sacco S, Volterrani D, Di Luca L, Buralli S, Di Stefana R, Baranowska-Kartylewicz J, Bonara D, Matteucci F, Ricci S, Bellina CR, Falcone A, Salvadori PA, Mosca F, Adelstein SJ and Kassis AI (1996*b*) Tumour targeting by intra-arterial infusion of 5-[$^{123}$I]iodo-2′-deoxyuridine in patients with liver metastases from colorectal cancer. *J Nucl Med* **37** (suppl.): 22S–25S.

Neshasteh-Riz A, Angerson WJ, Reeves JR, Smith G, Rampling R and Mairs RJ (1997) Incorporation of iododeoxiuridine in multicellular spheroids: implications for DNA-targeted radiotherapy using Auger electron emitter. *Br J Cancer* **75**: 493–499

O'Donoghue JA, Bardies M and Wheldon TE (1995) Relationships between tumour size and curability for uniformly targeted therapy with beta-emitting radionuclides. *J Nucl Med* **36**: 1902–1909

Raaphorst GP, Feeley MMDa, Silva VfDanjoux CE and Gerig LH (1989) A comparison of heat and radiation sensitivity of three human glioma cell lines. *In J Radiat Oncol Biol Phys* **17**: 615–622

Rodriguez R, Ritter M, Fowler JF and Kinsella TJ (1994) Kinetics of cell labelling and thymidine replacement after continuous infusion of halogenated pyrimidines *in vivo*. Int J Radiat Oncol Biol Phys **29**: 105–113

Schneiderman MH and Schneiderman GS (1996) Radioiododeoxyuridine in cancer therapy: an in vitro approach to developing in vivo strategies. *J Nucl Med* **37** (suppl.): 6S–9S

Schwartz JL, Mustafi R, Hughes A and Desomber ER (1996) DNA and chromosome breaks by iodine-123 labelled estrogen in chinese-hamster ovary cells. *Radiat Res* **146**: 151–158

Sutherland RM (1988) Cell environment interactions in tumour microregions – the multicell spheroid model. *Science* **240**: 177–184

Taghian A, Suit H, Phil D, Pardo F, Gioioso D, Tomkinson K, DuBois W and Gerweck L (1992) In vitro intrinsic radiation sensitivity of glioblastoma multiforme. *Int J Radiat Oncol Biol Phys* **23**: 55–62

Vaidyanathan G, Larsen RH and Zalutsky MR (1996) 5-[$^{211}$At]astato-2′-deoxyuridine, an alpha particle-emitting endoradiotherapeutic agent undergoing DNA incorporation. *Cancer Res* **56**: 1204–1209

Wheldon TE and O'Donoghue JA (1990) The radiobiology of targeted radiotherapy. *Int J Radiat Biol* **58**: 1–21

© Cancer Research Campaign 1998